# A multi-year heavy metal analysis of 72 dark chocolate and cocoa products in the USA

**DOI:** 10.3389/fnut.2024.1366231

**Published:** 2024-07-31

**Authors:** Jacob M. Hands, Mark L. Anderson, Tod Cooperman, Jared E. Balsky, Leigh A. Frame

**Affiliations:** ^1^The George Washington University School of Medicine and Health Sciences, Washington, DC, United States; ^2^Department of Research, ConsumerLab.com, White Plains, NY, United States; ^3^Integrative Medicine, The George Washington University School of Medicine and Health Sciences, Washington, DC, United States; ^4^Resiliency and Well-Being Center, The George Washington University School of Medicine and Health Sciences, Washington, DC, United States

**Keywords:** lead, cadmium, metal, cocoa, cocoa and chocolates

## Abstract

**Introduction:**

Contamination of cocoa containing products, such as dark chocolate, with heavy metals including lead, cadmium and arsenic has been reported in the US. However, a formal exploration into the significance of this contamination, nor multi-year trends in the degree or scope remain unresolved.

**Methods:**

From 2014 to 2022, 72 consumer cocoa-containing products were purchased and analyzed for heavy metal contamination with lead (Pb), cadmium (Cd), and arsenic (As) in 4 distinct cohorts (2014, 2016, 2019, 2022). The thresholds used to assess heavy metal contamination were set to Prop 65 maximum allowable dose levels (MADLs) of 0.5 mcg/day, 4.1 mcg/day, 10 mcg/day for Pb, Cd, and As, respectively.

**Results and discussion:**

Our analysis reports that 43, 35, and 0% of the products tested exceed Prop 65 MADLs for heavy metal concentrations, respectively, of Pb, Cd, and As, while 97.2% (70 of 72) fall below US FDA IRL limits established for Pb. Median concentrations of each metal tested were lower than even the conservative Prop 65 MADLs, indicating a potentially large effect of product outliers. This indicates that heavy metal contamination—in more than half of products tested—may not pose any appreciable risk for the average person when consumed as a single serving; however, consuming some of the products tested, or more than one serving per day in combination with non-cocoa derived sources heavy metals, may add up to exposure that would exceed the Prop 65 MADLs. Notably, “organic” products were significantly more likely to demonstrate higher levels of both Cd and Pb.

## Introduction

The contamination of consumer cocoa-containing products such as dark chocolate with heavy metals, including lead (Pb), cadmium (Cd), and arsenic (As), has been reported by prominent consumer media outlets and independent testing agencies (1–4). Such findings may significantly alter consumer dietary behaviors, as they may opt against the consumption of cocoa-containing products owing to purported health risks associated with heavy metal contamination. The ramifications nor the clinical veracity of these claims have been explored in the literature. Recent research has further underscored the global concern regarding heavy metal contamination in food products, highlighting the need for stringent monitoring and regulation (5).

Heavy metal contamination in consumer foods remains a significant issue globally, though there is evidence of progress in the domestic United States (US), with serum levels of heavy metals, particularly Pb, falling by almost 95% since 1976 (6–10). Heavy metal contamination has been demonstrated to be pervasive across nations and has been detected in nearly every food category from grain to meat-products, with vegetable and cereal products most affected by industrial and environmental sources of contamination (11–16). Pb, Cd, As and Hg are typically evaluated for contamination as they represent the most common health threats to consumers (9–23). National initiatives, including the US Food and Drug Administration (FDA) Closer to Zero Program (C2Z), have been implemented and aim to reduce the net level of contamination in foods, especially those consumed by sensitive groups such as toddlers and expectant mothers, to the lowest possible level while maintaining access to nutritious foods (24). The C2Z works by establishing an interim reference level (IRL) or maximum allowable intake corresponding to a serum target roughly 10-fold below the accepted reference level or value for a particular toxicant. In the case of Pb, for example, the IRL is derived from the blood Pb reference value (BLRV) or the 97.5% of blood lead levels (BLLs) in children 1–5 years of age identified during US Center for Disease Control (CDC) surveillance of the National Health and Nutrition Examination Survey (NHANES). As of 2022, the IRL for has been amended to 2.2 mcg/day for children under 7 years of age and 8.8 mcg/day of Pb for women of child-bearing age (24, 25). This is a 64% decrease from the previous cutoff for Pb of 6 mcg/day for young children and 25 μg/day for pregnant or lactating women in the early 1990’s, but this is still well above the California Proposition 65 (Prop 65) maximum allowable dose levels (MADLs).

While federal regulations pertaining to consumer Pb contamination have received, perhaps, the most visibility, proposed reference ranges have been offered by several agencies for both As and Cd. MADLs set on daily consumption by Prop 65 mandate that total Cd and As consumption be 4.1 and 10 mcg, respectively (26–28).

Cocoa-containing products are notoriously rich in metals owing to cultivation and manufacturing practices required to produce such products (29). Dark chocolate in particular is a rich source of earth metals and contamination in the course of its processing has been well documented. Specifically, it is thought that the proximity of cocoa-farms and processing plants apparent proximity to other processing and industrial facilities, which have been demonstrated to be significant sources of pollution, is a driver of heavy metal contamination in dark chocolate. While several authors have noted that heavy metal contamination of dark chocolate and other cocoa products is chiefly the result of post harvesting contamination, the precise level of contamination and the clinical implications of this contamination for domestic dark chocolate and cocoa-containing products is largely unexplored in the literature (6, 30).

Our aim is to assess the heavy metal contamination of the most popular cocoa-containing consumer products each year for several years to assess trends. In this study, we report the findings from a multi-year heavy metal contamination survey (2014–2022) of 72 cocoa-containing products purchased in the domestic US, which were identified through annual consumer surveys of product popularity. This data serves to provide a window into the extent of the contamination problem in US cocoa-containing products such as dark chocolate.

## Methods

From 2014 to 2022, 72 consumer cocoa-containing products were purchased from retail sources and analyzed for heavy metal contamination with Pb, Cd, and As in 4 distinct cohorts (2014, 2016, 2019, 2022). Pb, Cd and As were selected as they tend to be the most common sources of heavy metals in cocoa containing products owing to their presence in soil and their role as industrial waste products in and around cocoa processing sites.

The thresholds used to assess heavy metal contamination were set to Prop 65 MADLs of 0.5 mcg/day, 4.1 mcg/day, 10 mcg for Pb, Cd, and As, respectively. All heavy metal concentrations are listed in mcg/g and were scaled to mcg/day based on listed servings per product and assuming one serving per day. Seals and certifications (e.g., Organic, Fairtrade, Non-GMO) were recorded.

All products were purchased by ConsumerLab.com from third party online retailers (e.g., Amazon, iHerb), physical retailers (e.g., GNC, Whole Foods) and manufacturer or distributor websites. All products were domestic or European produced and obtained domestically. Products were selected largely according to a yearly ranked product survey taken by 8,000–10,000 respondents through the online site, ConsumerLab.com, an independent testing company committed to providing transparency and clinically-guided knowledge of nutritional supplementation to consumers and healthcare providers. Product selection in each year was affected by previous findings: Products with higher levels of heavy metal contamination were often excluded from subsequent testing, consistent with ConsumerLab’s stated mission “To help consumers and healthcare professionals find the best quality health and nutrition products through independent testing and evaluation.”

Two independent commercial laboratories in the domestic US were enlisted by ConsumerLab.com for analysis employing acid or microwave digestion and Inductively Coupled Plasma-Mass Spectroscopy (ICP-MS). For the lead analysis, nominal 0.5 gram weights of the samples were digested with trace metals grade concentrated nitric acid, hydrochloric acid and hydrogen peroxide using a PerkinElmer Multiwave 3,000 Anton Paar Microwave Reaction System. After digestion, the samples were brought to a final volume of 50 mL with DI water. For arsenic and cadmium analysis, nominal 1 g weights of the samples were digested on a hotplate with trace metals grade concentrated nitric acid. After digestion, the samples were brought to a final volume of 200 mL with DI water. Samples were analyzed for metals on a PerkinElmer NexIon 350X ICPMS. Reporting limits were 0.01ug/g for lead and 0.02 ug/g for arsenic and cadmium. Analytic techniques used for testing of Pb, Cd and As in analyzed products are consistent with AOAC 2015.01 methods for the determination of heavy metal in food products. QC limits were 75%–125% for spiked recoveries. Calibration standards for arsenic were 0.001, 0.05, 0.1, and 0.5 ppm. Calibration standards for lead and cadmium were 0.0005, 0.002, 0.010, and 0.02 ppm. QC limits of variation 75%–125% and ±10% for second source continuing check standard of 0.1 ppm for arsenic and 0.01 ppm for lead and cadmium. Samples exceeding the pre-specified Prop 65 thresholds were retested at one of two independent laboratories for confirmation of heavy metal contamination.

A series of multivariate linear regressions with fixed and random effects were generated to evaluate the impact of ‘number of attained third party certifications’ and specific certifications (e.g., USDA Organic), controlling for cohort testing year, on total heavy metal concentrations for Pb, As, and Cd. Marginal linear predictions were derived and used to assess the impact of cohort year on heavy metal contamination. Bonferroni correction was applied in head-to-head marginal comparison. Hausman testing of endogeneity was used to determine the suitability of fixed vs. standard random effects. Robust standard errors were assumed. A multivariate Ordinary Least Squares model with robust standard errors was also generated and used as a robustness check to evaluate the effect of year on metal contamination. KW testing was employed to ascertain significant differences in the content of each metal by year of testing. Finally, Welch’s T-test with unequal standard errors was performed to better isolate the contribution of “organic” in the context of all metals. All statistics were calculated using StataBE 18 (Stata Corp, College Station, Tx).

## Results

**Figure 1:**
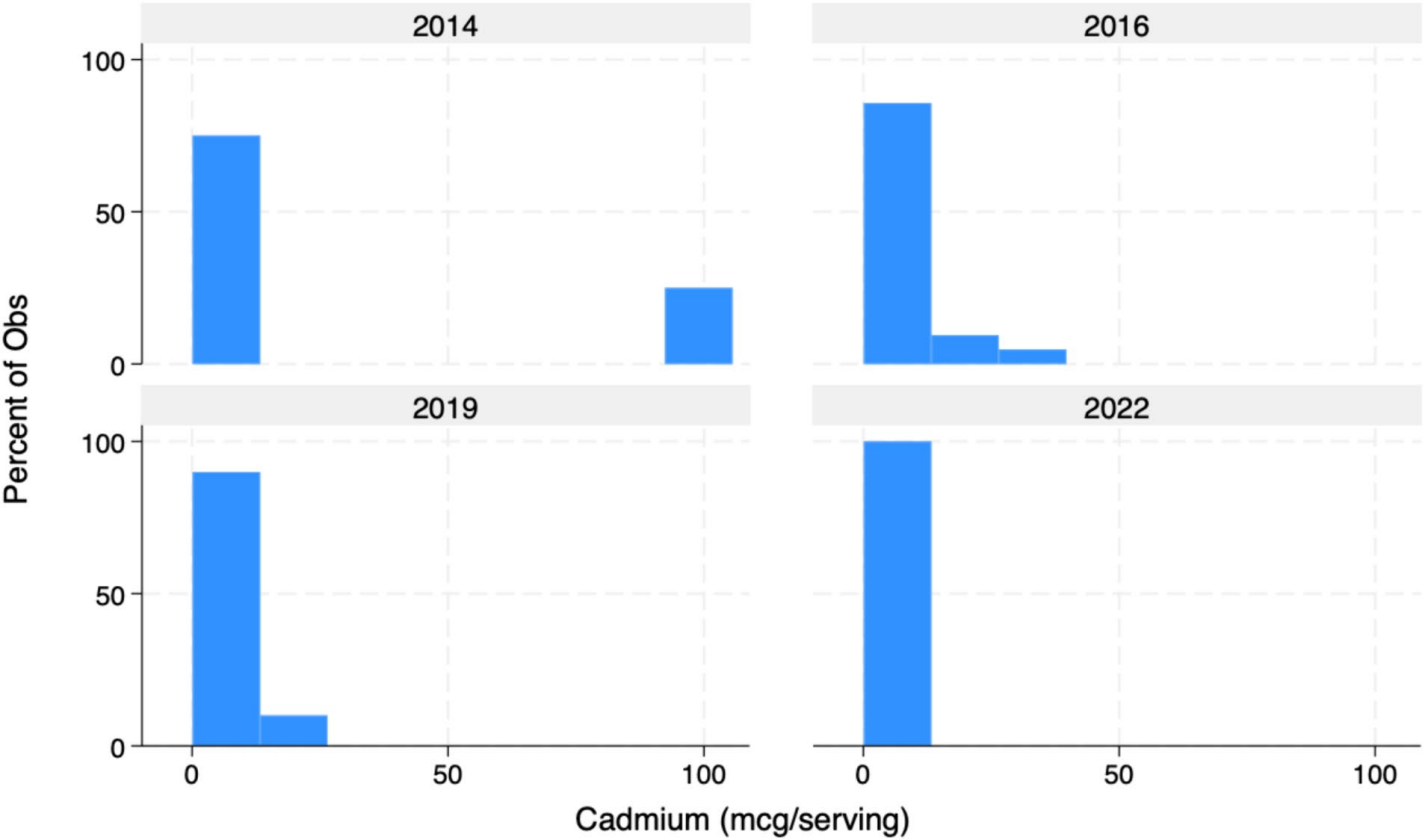
Cadmium (Cd) in mcg/serving of listed products (%). Graph of Cd concentrations detected in 72 samples. The presence of outliers in 2014 skews the apparent improvement of Cd contamination in cocoa products from 2014 to 2022.

**Figure 2:**
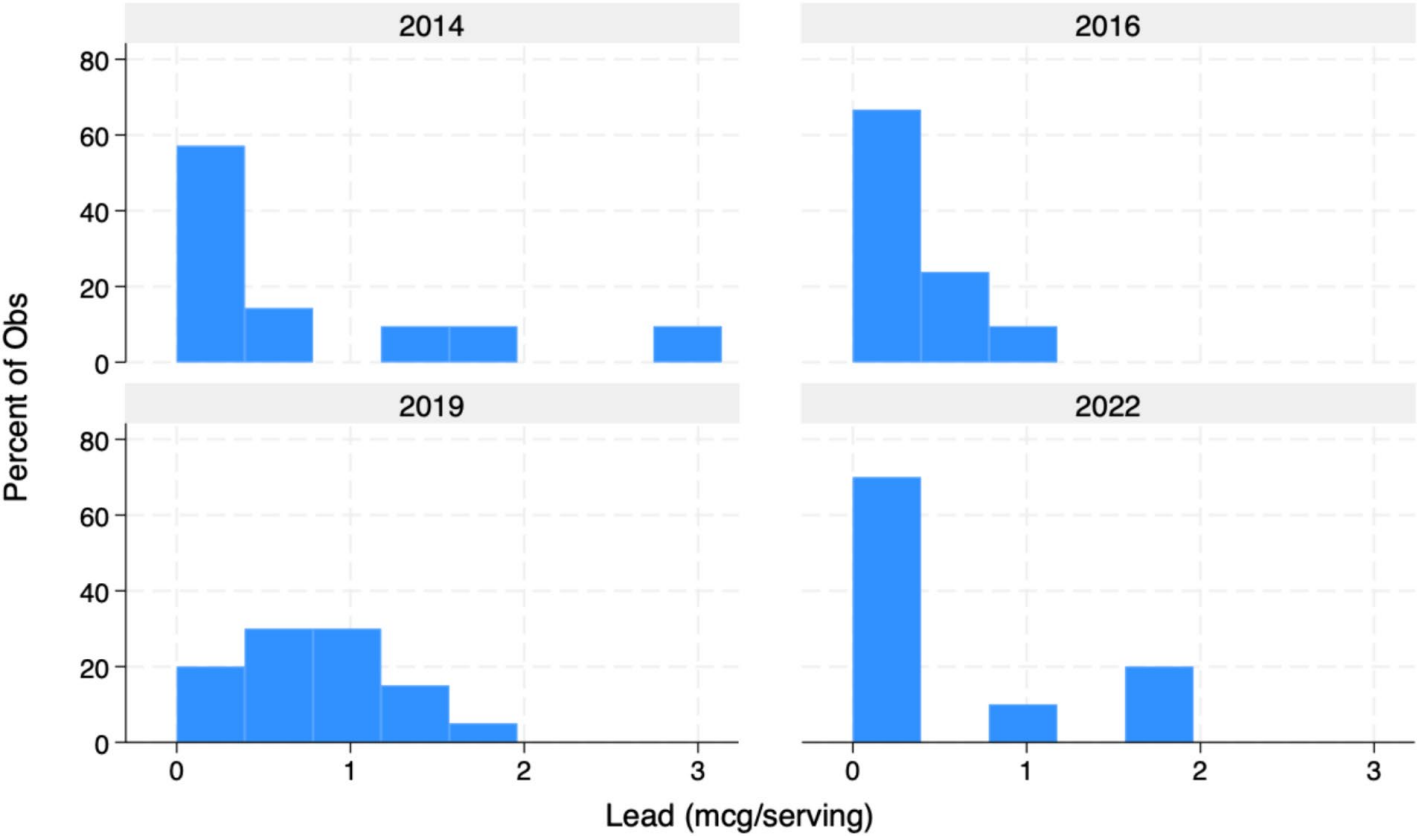
Lead [Pb in mcg/serving of listed products (%)]. Graph of Pb concentrations detected in 72 samples.

**Figure 3:**
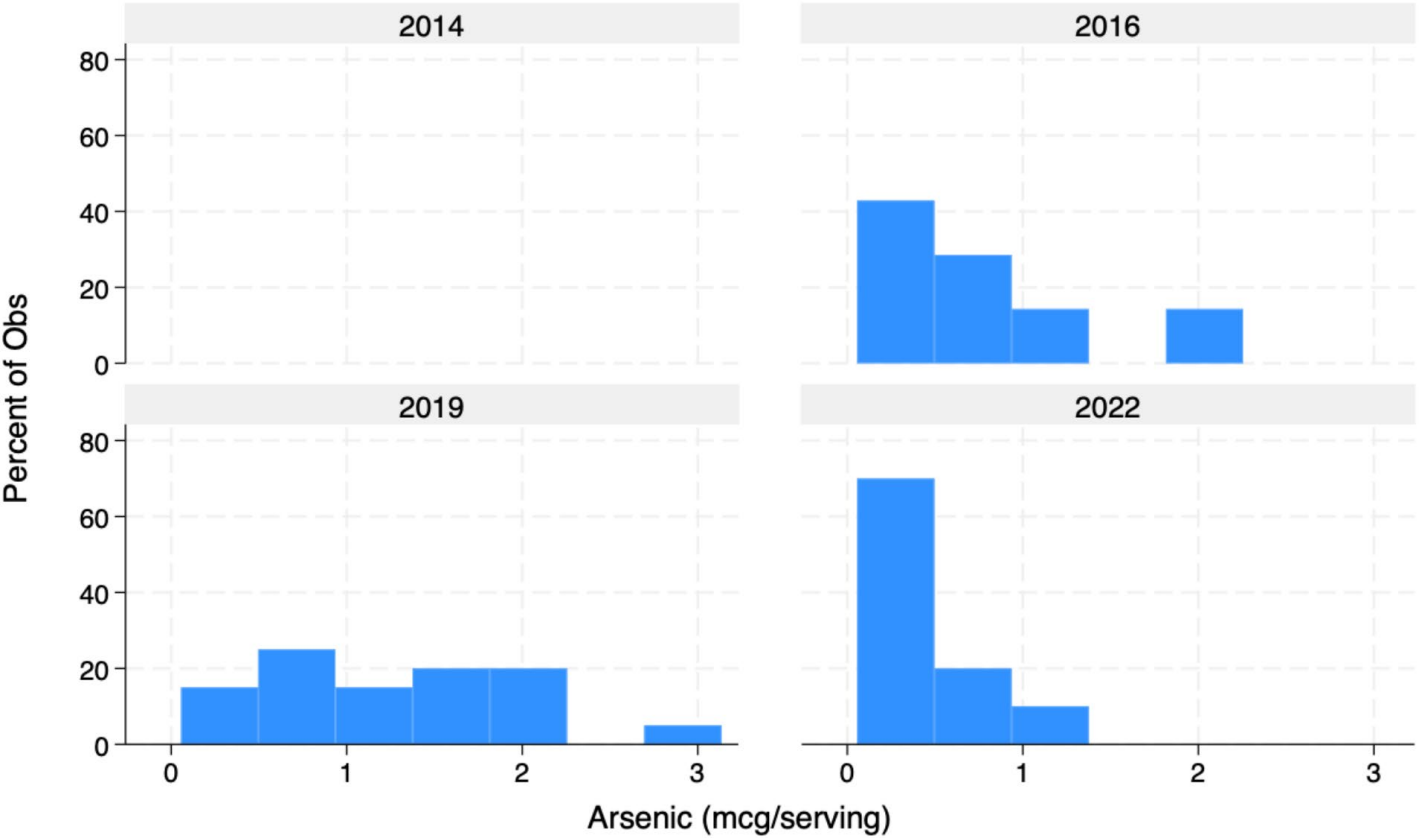
Arsenic in mcg/serving of listed products (%). Graph of As concentrations detected in 72 samples form 2014–2022. No sample exceeded As Prop 65 limits for contamination.

Overall, mean concentrations per listed serving of both Pb (0.615 mcg/serving) and Cd (4.358 mcg/serving) exceeded Prop 65 MADLs (0.5mcg/day and 4.1 mcg respectively) among all products tested (Table 1). Mean As concentrations (0.931) were universally compliant and well-below 10 mcg Prop 65 MADL. Among all products tested per serving, 31 of 72 (43%) exceeded Prop 65 limits for Pb, while 13 of 37 (35%) exceeded Prop 65 limits for Cd. Importantly, however, median concentrations of Pb (0.375 mcg/day) and Cd (3.03 mcg/day) tested below Prop 65 standards, indicating a potentially large effect associated with the inclusion of outliers (Figures 1–3). Of note, while FDA IRLs have not been established for Cd or As, mean concentrations of Pb still resulted in levels more than 12-fold lower than the relatively less conservative US federal limits for expectant mothers and nearly 4-fold lower than those set for children younger than 7 (7).

**Table 1:** Summary statistics.

	*N*	Mean	Median	CA Prop65 limit	Min	Max
Lead (mcg/g)	72	0.062	0.05	–	0.009	0.269
Lead (mcg/serving)	72	0.615	0.375	0.5	0	3.136
Lead (mcg/day)	72	0.615	0.375	0.5	0	3.136
Cadmium (mcg/g)	72	0.396	0.239	–	0.016	2.2
Cadmium (mcg/serving)	55	6.986	3.03	4.1	0.1	92.4
Cadmium (mcg day)	37	4.358	2.725	4.1	0.29	14.12
Arsenic (mcg/g)	55	0.094	0.05	–	0.017	0.2
Arsenic (mcg/serving)	37	0.931	0.75	10	0.056	2.695
Arsenic (mcg/day)	37	0.931	0.75	10	0.056	2.695
Lead (mcg/g) (2014)	17	0.098	0.1	–	0.025	0.269
Arsenic (mcg/g) (2014)	NA	NA	NA	–	NA	NA
Cadmium (mcg/g) (2014)	17	0.512	0.224	–	0.06	1.52
Lead (mcg/g) (2016)	17	0.06	0.05	–	0.05	0.141
Arsenic (mcg/g) (2016)	17	0.196	0.2	–	0.132	0.2
Cadmium (mcg/g) (2016)	17	0.593	0.288	–	0.1	2.2
Lead (mcg/g) (2019)	21	0.044	0.03	–	0.025	0.106
Arsenic (mcg/g) (2019)	21	0.058	0.05	–	0.037	0.2
Cadmium (mcg/g) (2019)	21	0.295	0.225	–	0.054	0.982
Lead (mcg/g) (2022)	17	0.05	0.055	–	0.009	0.113
Arsenic (mcg/g) (2022)	17	0.038	0.037	–	0.017	0.078
Cadmium (mcg/g) (2022)	17	0.208	0.24	–	0.016	0.521

Summary of mean, median concentrations of heavy metals from 2014 to 2022 for all available samples. Arsenic was not tested as mcg/g in 2014.

Neither trade certifications, nor “organic” designation, resulted in lower concentrations of Pb, Cd, or As (Tables 2, 3). Those products certified as organic were, unexpectedly, associated with a statistically significant 3.163 greater mcg/serving greater concentration of Cd per serving of cocoa or dark chocolate; though, this association was tenuous and varied by specification (Table 2). When controlling for the mcg/g concentration, however, products noted as “organic” were significantly more likely to display higher levels of Cd (0.4 mcg/g) and Pb (0.02 mcg/g; Table 3, *p* < 0.001). Welch’s T-testing demonstrated that organic products were not significantly more likely to present with elevated levels of Pb nor As. In contrast, Cd concentrations were again significantly greater in organic products [t = −2.1, Pr (T < t) = 0.02, Table 4].

**Table 2:** Panel regression with FEs robust SE.

	(1)	(2)	(3)
Variables	Lead (mcg/serving)	Cd (mcg/serving)	Arsenic (mcg/serving)
Organic	0.0263	3.163^**^	0.108^*^
	(0.194)	(0.335)	(0.0121)
Certifications (quantity)	0.0195	−0.674	−0.0659
	(0.109)	(0.928)	(0.0478)
Serving (g or mL)	0.0108	0.330	0.0364
	(0.00879)	(0.150)	(0.00718)
Observations	71	54	37
R-squared	0.070	0.142	0.686
Number of years tested	4	3	2

Multivariate linear regression of heavy metals from 2014 to 2022 for all available samples in mcg/serving. Several samples were dropped owing to one or more incomplete entries required for the model. Robust standard errors in parentheses. ^***^p < 0.01, ^**^p < 0.05, ^*^p < 0.1.

**Table 3:** OLS Regression with Robust SEs.

	(1)	(2)	(3)
Variables	Lead (mcg/gram)	Cd (mcg/gram)	Arsenic (mcg/gram)
Organic?	0.0202^***^	0.401^***^	0.0204
	(0.00715)	(0.148)	(0.0141)
Certifications (quantity)	−0.00896^***^	−0.0745^*^	−0.00432
Year (compared with 2014)	(0.00300)	(0.0415)	(0.00368)
2016	−0.0391^**^	0.0678	
	(0.0167)	(0.178)	
2018	−0.0492^***^	−0.198^*^	−0.136^***^
	(0.0161)	(0.102)	(0.00966)
2022	−0.0466^***^	−0.335^***^	−0.159^***^
	(0.0161)	(0.101)	(0.00624)
Observations	72	72	55
R-squared	0.260	0.240	0.900

Multivariate linear regression of heavy metals from 2014–2022 for all available samples. Arsenic was not tested as mcg/g in 2014. Robust standard errors in parentheses. ^***^p < 0.01, ^**^p < 0.05, ^*^p < 0.1.

**Table 4:** Two-sample Welch’s T-test for cadmium (mcg/day) for not organic and organic.

Group	Observations	Mean	Std. err.	Std. dev.	[95% conf. interval]
Not organic	22	3.176	0.835	3.914	1.44–4.91
Organic	15	6.09	1.064	4.123	3.80–8.38
Combined	37	4.36	0.690	31.5013	2.96–5.76
Diff	–	−2.91	1.352	–	−5.67–−0.157
					
T-stat	−2.1560	Pr (T < t)	0.0195	-	

As a whole, products in later cohorts (2016, 2019, 2022) demonstrated lower concentrations of all heavy metals tested as compared with those in 2014, with significantly lower concentrations of lead documented between the years 2022 vs. 2014 and the years 2019 vs. 2014 (Table 5). KW testing confirmed that year of testing was jointly associated with tested levels of Pb and As (*p* < 0.001).

**Table 5:** Pairwise comparison testing of heavy metals by year with BE correction to adjust for multiple comparisons from 2014 to 2022.

Year	Contrast	Bonferroni standard error	t	*P* > t
**Pb (mcg/g) pairwise comparison (batch testing by year)**				
2016 vs. 2014	−0.0391111	0.0166747	−2.35	0.132
2019 vs. 2014	**−0.0492426**	**0.0161305**	**−3.05**	**0.020**
2022 vs. 2014	**−0.046642**	**0.0161116**	**−2.89**	**0.031**
2019 vs. 2016	−0.0101315	0.0089885	−1.13	1.000
2022 vs. 2016	−0.007531	0.0091391	−0.82	1.000
2022 vs. 2019	0.0026005	0.0090927	0.29	1.000
**Cadmium (mcg/g) pairwise comparison (batch testing by year)**				
2016 vs. 2014	0.0677999	0.1781246	0.38	1.000
2019 vs. 2014	−0.1975056	0.1018891	−1.94	0. 341
2022 vs. 2014	**−0.3348801**	**0.1005712**	**−3.33**	**0.009**
2019 vs. 2016	−0.2653055	0.1506569	−1.76	0.497
2022 vs. 2016	−0.40268	0.1496009	−2.69	0.054
2022 vs. 2019	−0.1373745	0.0625568	−2.20	0.190
**As (mcg/g) pairwise comparison (batch testing by year)**				
2019 vs. 2016	−0.1360507	0.009663	−14.08	<0.0001
2022 vs. 2016	−0.1585439	0.0062441	−25.39	<0.0001
2022 vs. 2019	−0.0224932	0.0097177	−2.31	0.074

Bold values means Bonferroni correction.

## Discussion

We report the concentrations of Pb, Cd, and As for 72 popular cocoa-containing consumer products (e.g., dark chocolate) sampled over a period of 8 years.

Our analysis suggests that less than 50% of the products tested exceed Prop 65 MADLs for heavy metal concentrations of Pb and Cd, while 97.2% (70 of 72) fall below US FDA IRL limits established for Pb. Moreover, median concentrations of each metal tested were lower than even the conservative Prop 65 MADLs. This indicates that the heavy metal contamination, in the majority of products surveyed, may not pose a risk for the average person when consumed as a single serving; however, consuming more than one serving per day and/or in combination w other sources of heavy metals (e.g., seafood) may cumulatively add up to exposure that exceed the Prop 65 MADLs.

The question of “hazard” associated with consumer consumption of heavy metals contained in cocoa-containing products is complex. Taken at their mean or median concentrations, the risk associated with Pb consumption, for example, is “low” or almost 4-fold lower than the most stringent US FDA IRL of 2.2 mcg/day of Pb for children under 7 (30). Yet, significant outliers (maximum tested Pb sample = 3.13 mcg/serving) do, in fact, begin to exceed the parameters of conservative IRLs. Further, it should be remarked that there has never been a safe serum level of Pb consumption identified with any value, even those under the 3.5 mcg/day threshold (0.5 IQ points), are associated with adverse effects across a range of systems (30, 31). Indeed, prior authors have demonstrated that the steepness of the slope between IQ and BLL, for instance, may be more prominent at lower serum Pb levels. Thus, any intake of Pb, especially among vulnerable populations, should be heavily scrutinized.

Similarly, while there has yet to be a federal limit identified for Cd consumption, the concentrations found in several samples significantly exceeded multiple regulatory recommendations for safe limits (32, 33). The biological effects associated with Cd consumption, even at levels below regulatory standards, indicate that Cd levels are associated with cardiovascular disease, kidney dysfunction, cognitive deficit, diabetes, cancer, osteoporosis, and a panoply of related conditions (34–46). Therefore, like Pb, the maximum reasonable reduction of dietary exposure, especially for developing children and expectant mothers, is highly advisable.

Though the results of this analysis suggest that the overall consumer market may be, on average, without biologically significant contamination as individual servings, the presence of outliers as well as the potential additive exposure through other foods, such as teas and spices, is potentially problematic (17–23, 47). More interesting was the observation that “organic” products were significantly more likely to demonstrate higher levels of both Cd and Pb. More striking, the number of trade certifications (e.g., Non-GMO, Fairtrade) did not significantly alter the levels of heavy metals found among products surveyed. Thus, even those consumers opting to purchase “higher” quality products retained no exposure protection. Such heterogeneity invites potentially hazardous exposures, especially among biologically susceptible groups, necessitating enhanced surveillance. Cocoa-containing products are a rich source of dietary polyphenols namely flavonoids such as catechins and anthocyanidins, which may be beneficial to health, though the benefits associated with cocoa-containing products have been typically been modest (46, 48, 49, 50).

Since it has been documented that heavy metal contamination of dark chocolate and other cocoa products is chiefly the result of post harvesting contamination, better quality control practices during harvesting and manufacturing may help eliminate the problem.

## Conclusion

The results of our analysis suggest that many products contain Pb and Cd in amounts that may exceed certain, stringent regulatory requirements (Prop 65). Therefore, enhanced surveillance may be warranted. Further, additional research into cumulative heavy metal exposure from the diet as a whole would help put this work into context to best inform public health policy and interventions. For instance, if contaminated products as a whole are consumed in small amounts and infrequently by most, these contaminants may not be a public health concern (though, perhaps still an individual concern); in contrast, if many such products are consumed fairly regularly by the average consumer, the additive exposure may be a public health concern. However, high mean contamination and the presence of outliers with greater contamination should motivate greater testing of all consumer products, particularly cocoa, to better address significant gaps in quality control. Additionally, this must be placed in the context of dietary patterns and public health concern.

## Data availability statement

The raw data supporting the conclusions of this article will be made available by the authors, without undue reservation.

## Author contributions

JH: Conceptualization, Data curation, Formal analysis, Funding acquisition, Investigation, Methodology, Project administration, Resources, Software, Supervision, Validation, Visualization, Writing – original draft, Writing – review & editing. MA: Conceptualization, Data curation, Formal analysis, Funding acquisition, Investigation, Methodology, Project administration, Resources, Software, Supervision, Validation, Visualization, Writing – original draft, Writing – review & editing. TC: Conceptualization, Data curation, Formal analysis, Investigation, Methodology, Project administration, Resources, Supervision, Validation, Visualization, Writing – original draft, Writing – review & editing. JB: Conceptualization, Data curation, Formal analysis, Investigation, Methodology, Project administration, Resources, Supervision, Validation, Visualization, Writing – original draft, Writing – review & editing. LF: Conceptualization, Data curation, Formal analysis, Investigation, Methodology, Project administration, Resources, Software, Supervision, Validation, Visualization, Writing – original draft, Writing – review & editing.

## References

[1] Consumer Reports. Available at: https://www.consumerreports.org/health/food-safety/lead-and-cadmium-in-dark-chocolate-a8480295550/ (n.d.).

[2] Abt E Fong Sam J Gray P Robin LP. Cadmium and lead in cocoa powder and chocolate products in the US market. Food Addit Contam Part B. (2018) 11:92–102. doi: 10.1080/19393210.2017.1420 29310543

[3] Karaś K Zioła-Frankowska A Bartoszewicz M Krzyśko G Frankowski M. Investigation of chocolate types on the content of selected metals and non-metals determined by ICP-OES analytical technique. Food Addit Contam Part A. (2020) 38:293–303. doi: 10.1080/19440049.2020.1853 33332983

[4] Lo Dico GM Galvano F Dugo G D’ascenzi C Macaluso A Vella A. Toxic metal levels in cocoa powder and chocolate by ICP-MS method after microwave-assisted digestion. Food Chem. (2018) 245:1163–8. doi: 10.1016/j.foodchem.2017.11 29287336

[5] Daher Z el Deghel N al Habahbeh R Azoury M. Cadmium levels in locally produced and imported dark chocolate in Lebanon. Expo Health. (2023). doi: 10.1007/s12403-023-00614-4

[6] Taylor DA. Lead in cocoa products: where does contamination come from? Environ Health Perspect. (2005) 113:A687–8. doi: 10.1289/ehp.113-a687

[7] Rankin CW Nriagu JO Aggarwal JK Arowolo TA Adebayo K Flegal AR. Lead contamination in cocoa and cocoa products: isotopic evidence of global contamination. Environ Health Perspect. (2005) 113:1344–8. doi: 10.1289/ehp.8009 16203244 PMC1281277

[8] Scutarașu EC Trincă LC. Heavy metals in foods and beverages: global situation, health risks and reduction methods. Food Secur. (2023) 12:3340. doi: 10.3390/foods12183340 PMC10528236 37761050

[9] Wang X Han X Guo S Ma Y Zhang Y. Associations between patterns of blood heavy metal exposure and health outcomes: insights from NHANES 2011–2016. BMC Public Health. (2024) 24:558. doi: 10.1186/s12889-024-17754-0, PMID: 38389043 PMC10882930

[10] Egan KB Cornwell CR Courtney JG Ettinger AS. Blood Lead Levels in U.S. children ages 1-11 years, 1976-2016. Environ Health Perspect. (2021) 129:37003. doi: 10.1289/EHP7932 33730866 PMC7969125

[11] Liang G Gong W Li B Zuo J Pan L Liu X. Analysis of heavy metals in foodstuffs and an assessment of the health risks to the general public via consumption in Beijing, China. Int J Environ Res Public Health. (2019) 16:909. doi: 10.3390/ijerph16060909 30871239 PMC6465990

[12] Koch W Czop M Iłowiecka K Nawrocka A Wiącek D. Dietary intake of toxic heavy metals with major groups of food products-results of analytical determinations. Nutrients. (2022) 14:1626. doi: 10.3390/nu14081626 35458187 PMC9029343

[13] Rai PK Lee SS Zhang M Tsang YF Kim KH. Heavy metals in food crops: health risks, fate, mechanisms, and management. Environ Int. (2019) 125:365–85. doi: 10.1016/j.envint.2019.01.067 30743144

[14] Rusin M Domagalska J Rogala D Razzaghi M Szymala I. Concentration of cadmium and lead in vegetables and fruits. Sci Rep. (2021) 11:11913. doi: 10.1038/s41598-021-91554-z, PMID: 34099845 PMC8184968

[15] Morgan JN. Effects of processing of heavy metal content of foods. Adv Exp Med Biol. (1999) 459:195–211. doi: 10.1007/978-1-4615-4853-9_13 10335377

[16] Collado-López S Betanzos-Robledo L Téllez-Rojo MM Lamadrid-Figueroa H Reyes M Ríos C. Heavy metals in unprocessed or minimally processed foods consumed by humans worldwide: a scoping review. Int J Environ Res Public Health. (2022) 19:8651. doi: 10.3390/ijerph19148651, PMID: 35886506 PMC9319294

[17] Mousavi Khaneghah A Mahmudiono T Javanmardi F Tajdar-oranj B Nematollahi A Pirhadi M. The concentration of potentially toxic elements (PTEs) in the coffee products: a systematic review and meta-analysis. Environ Sci Pollut Res Int. (2022) 29:78152–64. doi: 10.1007/s11356-022-23110-9, PMID: 36178656

[18] Salmani MH Gholami M Ranjbar MJ Mokhberi F. Comparison of essential and toxic metals levels in some herbal teas: a systematic review. Biol Trace Elem Res. (2024) 202:615–23. doi: 10.1007/s12011-023-03698-w 37198356

[19] Sarker A Kim JE Islam ARMT Bilal M Rakib MRJ Nandi R. Heavy metals contamination and associated health risks in food webs-a review focuses on food safety and environmental sustainability in Bangladesh. Environ Sci Pollut Res Int. (2022) 29:3230–45. doi: 10.1007/s11356-021-17153-7, PMID: 34739668 PMC8569293

[20] Vasconcelos Neto MC Silva TBC Araújo VE Souza SVC. Lead contamination in food consumed and produced in Brazil: systematic review and meta-analysis. Food Res Int. (2019) 126:108671. doi: 10.1016/j.foodres.2019.108671 31732043

[21] Einolghozati M Talebi-Ghane E Khazaei M Mehri F. The level of heavy metal in fresh and processed fruits: a study Meta-analysis, systematic review, and health risk assessment. Biol Trace Elem Res. (2023) 201:2582–96. doi: 10.1007/s12011-022-03332-1 35727404

[22] Naimi N Pilevar Z Ranaei V Mahmudiono T Fakhri Y Paseban A. The concentration of potentially toxic elements (PTEs) in apple fruit: a global systematic review, meta-analysis, and health risk assessment. Environ Sci Pollut Res Int. (2022) 29:54013–24. doi: 10.1007/s11356-022-21158-1, PMID: 35648350

[23] Abdulai PM Sam K Onyena AP Ezejiofor AN Frazzoli C Ekhator OC. Persistent organic pollutants and heavy metals in Ghanaian environment: a systematic review of food safety implications. Environ Monit Assess. (2024) 196:376. doi: 10.1007/s10661-024-12500-w, PMID: 38492071

[24] FDA. Available at: https://www.fda.gov/food/environmental-contaminants-food/closer-zero-reducing-childhood-exposure-contaminants-foods#:~:text=Closer%20to%20Zero%20uses%20a,of%20a%20contaminant%20is%20unavoidable (2024).

[25] Flannery BM Middleton K. Updated interim reference levels for dietary lead to support FDA’s closer to zero action plan. Regul Toxicol Pharmacol. (2022) 133:105202–2. doi: 10.1016/j.yrtph.2022.105202 35690180

[26] Wilhelm M Schulz C Schwenk M. Revised and new reference values for arsenic, cadmium, lead, and mercury in blood or urine of children: basis for validation of human biomonitoring data in environmental medicine. Int J Hyg Environ Health. (2006) 209:301–5. doi: 10.1016/j.ijheh.2006.01.004 16739256

[27] Schaefer HR Flannery BM Crosby LM Pouillot R Farakos SMS van Doren JM. Reassessment of the cadmium toxicological reference value for use in human health assessments of foods. Regul Toxicol Pharmacol. (2023) 144:105487. doi: 10.1016/j.yrtph.2023.105487, PMID: 37640100

[28] Wong C Roberts SM Saab IN. Review of regulatory reference values and background levels for heavy metals in the human diet. Regul Toxicol Pharmacol. (2022) 130:105122. doi: 10.1016/j.yrtph.2022.105122 35090957

[29] As You Sow. Expert investigation related to cocoa and chocolate products. (2023). Available at: https://www.asyousow.org/reports/expert-investigation-related-to-cocoa-and-chocolate

[30] Flannery BM Dolan LC Hoffman-Pennesi D Gavelek A Jones OE Kanwal R. U.S. Food and Drug Administration's interim reference levels for dietary lead exposure in children and women of childbearing age. Regul Toxicol Pharmacol. (2020) 110:104516. doi: 10.1016/j.yrtph.2019.104516 31707132

[31] Heidari S Mostafaei S Razazian N Rajati M Saeedi A Rajati F. The effect of lead exposure on IQ test scores in children under 12 years: a systematic review and meta-analysis of case-control studies. Syst Rev. (2022) 11:106. doi: 10.1186/s13643-022-01963-y, PMID: 35637522 PMC9150353

[32] Genchi G Sinicropi MS Lauria G Carocci A Catalano A. The effects of cadmium toxicity. Int J Environ Res Public Health. (2020) 17:3782. doi: 10.3390/ijerph17113782 32466586 PMC7312803

[33] Rafati Rahimzadeh M Rafati Rahimzadeh M Kazemi S Moghadamnia AA. Cadmium toxicity and treatment: an update. Caspian J Intern Med. (2017) 8:135–45. doi: 10.22088/cjim.8.3.135 28932363 PMC5596182

[34] Tellez-Plaza M Jones MR Dominguez-Lucas A Guallar E Navas-Acien A. Cadmium exposure and clinical cardiovascular disease: a systematic review. Curr Atheroscler Rep. (2013) 15:356. doi: 10.1007/s11883-013-0356-2 23955722 PMC3858820

[35] Aramjoo H Arab-Zozani M Feyzi A Naghizadeh A Aschner M Naimabadi A. The association between environmental cadmium exposure, blood pressure, and hypertension: a systematic review and meta-analysis. Environ Sci Pollut Res Int. (2022) 29:35682–706. doi: 10.1007/s11356-021-17777-9, PMID: 35257333

[36] Doccioli C Sera F Francavilla A Cupisti A Biggeri A. Association of cadmium environmental exposure with chronic kidney disease: a systematic review and meta-analysis. Sci Total Environ. (2023) 2023:167165. doi: 10.1016/j.scitotenv.2023.167165 37758140

[37] Liu Z Cai L Liu Y Chen W Wang Q. Association between prenatal cadmium exposure and cognitive development of offspring: a systematic review. Environ Pollut. (2019) 254:113081. doi: 10.1016/j.envpol.2019.113081 31473391

[38] Chatterjee M Kortenkamp A. Cadmium exposures and deteriorations of cognitive abilities: estimation of a reference dose for mixture risk assessments based on a systematic review and confidence rating. Environ Health. (2022) 21:69. doi: 10.1186/s12940-022-00881-9 35836177 PMC9281031

[39] Filippini T Wise LA Vinceti M. Cadmium exposure and risk of diabetes and prediabetes: a systematic review and dose-response meta-analysis. Environ Int. (2022) 158:106920. doi: 10.1016/j.envint.2021.106920 34628255

[40] Filippini T Torres D Lopes C Carvalho C Moreira P Naska A. Cadmium exposure and risk of breast cancer: a dose-response meta-analysis of cohort studies. Environ Int. (2020) 142:105879. doi: 10.1016/j.envint.2020.105879, PMID: 32599354

[41] Nagaraju R Kalahasthi R Balachandar R Bagepally BS. Cadmium exposure and DNA damage (genotoxicity): a systematic review and meta-analysis. Crit Rev Toxicol. (2022) 52:786–98. doi: 10.1080/10408444.2023.2173557 36802997

[42] Cheng X Niu Y Ding Q Yin X Huang G Peng J. Cadmium exposure and risk of any fracture: a PRISMA-compliant systematic review and Meta-analysis. Medicine. (2016) 95:e2932. doi: 10.1097/MD.0000000000002932, PMID: 26962791 PMC4998872

[43] Kunioka CT Manso MC Carvalho M. Association between environmental cadmium exposure and osteoporosis risk in postmenopausal women: a systematic review and Meta-analysis. Int J Environ Res Public Health. (2022) 20:485. doi: 10.3390/ijerph20010485 36612804 PMC9820024

[44] Guo ZL Wang JY Gong LL Gan S Gu CM Wang SS. Association between cadmium exposure and urolithiasis risk: a systematic review and meta-analysis. Medicine. (2018) 97:e9460. doi: 10.1097/MD.0000000000009460 29505519 PMC5943130

[45] Habibian Sezavar A Abyareh M Fahimi R Nyasulu PS Abyadeh M. The association between maternal cadmium exposure and small for gestational age: a systematic review and meta-analysis. Int J Environ Health Res. (2022) 32:1469–77. doi: 10.1080/09603123.2021.1892035 33656412

[46] Gao X Li G Pan X Xia J Yan D Xu Y. Environmental and occupational exposure to cadmium associated with male reproductive health risk: a systematic review and meta-analysis based on epidemiological evidence. Environ Geochem Health. (2023) 45:7491–517. doi: 10.1007/s10653-023-01719-0, PMID: 37584848

[47] Huang Q Braffett BH Simmens SJ Young HA Ogden CL. Dietary polyphenol intake in US adults and 10-year trends: 2007-2016. J Acad Nutr Diet. (2020) 120:1821–33. doi: 10.1016/j.jand.2020.06.016 32807722

[48] Williamson G. The role of polyphenols in modern nutrition. Nutr Bull. (2017) 42:226–35. doi: 10.1111/nbu.12278 28983192 PMC5601283

[49] Tan TYC Lim XY Yeo JHH Lee SWH Lai NM. The health effects of chocolate and cocoa: a systematic review. Nutrients. (2021) 13:2909. doi: 10.3390/nu13092909 34578786 PMC8470865

[50] del Bo’ C Bernardi S Marino M Porrini M Tucci M Guglielmetti S. Systematic review on polyphenol intake and health outcomes: is there sufficient evidence to define a health-promoting polyphenol-rich dietary pattern? Nutrients. (2019) 11:1355. doi: 10.3390/nu11061355 31208133 PMC6627994

